# Probiotics as Additives on Therapy in Allergic Airway Diseases: A Systematic Review of Benefits and Risks

**DOI:** 10.1155/2013/231979

**Published:** 2013-07-15

**Authors:** Rashmi Ranjan Das, Sushree Samiksha Naik, Meenu Singh

**Affiliations:** ^1^Department of Pediatrics, All India Institute of Medical Sciences, Bhubaneswar 751019, India; ^2^Department of Obstetrics & Gynecology, SCB Medical College, Cuttack 753007, India; ^3^Department of Pediatrics, Postgraduate Institute of Medical Education and Research, Chandigarh 160012, India

## Abstract

*Background*. We conducted a systematic review to find out the role of probiotics in treatment of allergic airway diseases.  *Methods*. A comprehensive search of the major electronic databases was done till March 2013. Trials comparing the effect of probiotics versus placebo were included. A predefined set of outcome measures were assessed. Continuous data were expressed as standardized mean difference with 95% CI. Dichotomous data were expressed as odds ratio with 95% CI. *P* value < 0.05 was considered as significant. *Results*. A total of 12 studies were included. Probiotic intake was associated with a significantly improved quality of life score in patients with allergic rhinitis (SMD −1.9 (95% CI −3.62, −0.19); *P* = 0.03), though there was a high degree of heterogeneity. No improvement in quality of life score was noted in asthmatics. Probiotic intake also improved the following parameters: longer time free from episodes of asthma and rhinitis and decrease in the number of episodes of rhinitis per year. Adverse events were not significant. *Conclusion*. As the current evidence was generated from few trials with high degree of heterogeneity, routine use of probiotics as an additive on therapy in subjects with allergic airway diseases cannot be recommended.

## 1. Introduction

Allergy-related respiratory diseases include allergic rhinitis (AR), sinusitis and asthma. Concerning the T-helper (Th) cell (Th1/Th2) balance, it has been proposed that marked skewing of the immune response to Th2 lineage can result in an allergic disorder [1, 2]. Th2 polarization in allergic subjects may occur as a consequence of reduced pressure of microbial agents in the gut: the so-called “hygiene hypothesis” [3]. Probiotics are live microorganisms that, when administered in adequate amounts, confer a health benefit on the host. Probiotics may stimulate immune system at all mucosal surfaces and exert a primary prevention of atopic diseases and reduce allergic symptoms and inflammatory parameters [4]. Probiotics have been demonstrated to have anti-inflammatory properties associated with changes in cytokine expression that could potentially facilitate Th1 immune response, which could inhibit the development of allergic Th2 response and allergic antibody (IgE) production [5, 6]. As a result, they have been formally investigated in a number of clinical trials for the prevention and treatment of allergic respiratory diseases including asthma and AR, with some trials showing beneficial effects, but others not. This prompted us to conduct the current review to find whether the use of probiotics really benefits subjects with asthma and AR or not.

A recent Cochrane review done on preventive role of probiotics in allergic diseases (asthma, rhinitis, eczema, food allergy) has shown that there is insufficient evidence to recommend probiotics for prevention of allergic diseases [7]. In this review, five randomized controlled trials (RCTs) that compared the use of a probiotic to no probiotic were included. Three studies enrolling 1477 infants in the first six months of life without clinical evidence of allergic diseases, both with and without risk factors for allergy, were analysed. On final analysis, there was no significant difference in the incidence and prevalence of allergic diseases in both groups. As against this review, we have focused on the therapeutic role of probiotics given independently or as an adjunctive to drug treatment in subjects of all age groups and of both sexes with asthma and/or AR.

## 2. Methods

### 2.1. Criteria for Considering Studies for This Review

#### 2.1.1. Types of Studies

Randomized double-blind placebo-controlled trials (RCTs). 

#### 2.1.2. Types of Participants

Participants in trials were of either gender and of any age excluding infancy. Allergic asthma was defined by clinical history suggestive of bronchial hyperreactivity and demonstration of reversible airflow obstruction along with compatible positive prick tests to aeroallergens. The distinction of mild, moderate, severe intermittent, and/or persistent asthma was based on the Global Initiative for Asthma (GINA) guidelines [22]. Allergic rhinitis was defined by intermittent or continuous nasal symptoms for more than 1 yr along with either positive skin testing or specific IgE level against allergens. The distinction of mild, intermittent, persistent, and moderate-severe AR was based on the Allergic Rhinitis and its Impact on Asthma (ARIA) guidelines [23]. 

#### 2.1.3. Types of Interventions

Interventions consisted of daily treatment with probiotics or placebo (no probiotic bacteria), administered at the beginning of the study and continued for a minimum of >2 weeks, as an additive to standard antiallergic medications. All formulations of probiotics (irrespective of the type, strain and concentration) were considered.

#### 2.1.4. Types of Outcome Measures

Outcome measures frequently used to determine the clinical efficacy of any asthma or rhinitis treatment are quality of life score at the end of the treatment period, number of episodes, time free from episodes, participant/parent rated global assessment of treatment efficacy at the end of the treatment period, or percentage of symptom-free days during the treatment period. Accordingly, trials measuring following outcomes were included in the review.

- Primary outcome
  - Quality of life score at the end of treatment in AR or asthma.
- Secondary outcomes
  - Time (months) free from episodes of asthma or AR.
  - Mean duration of an episode of asthma or AR.
  - Number of episodes per year of asthma or AR.
  - Change in allergic lung/rhinitis symptom score (weekly).
  - Change in allergy and asthma medication score (weekly).
  - Cumulative number of asthma and rhinitis episodes.
  - Time (months) free from episodes of asthma/rhinitis.
  - Change in the pulmonary function tests.
  - Changes in blood parameters/immunological markers.
  - Side effects noted (if any).

Time or duration was defined as number of days for resolution of specific outcome from initiation of treatment. Change in symptom and/or medication score was defined as the change in total score over days per week. If the data were not available in the required format, the authors were contacted for the information. 

### 2.2. Search Methods for Identification of Studies

We systematically searched Medline, Cochrane Central Register of Controlled Trials (CENTRAL), EMBASE, and previous reviews including cross-references (all articles referenced), abstracts, and conference proceedings for all relevant articles till March 2013. The following keywords were used for retrieval of relevant articles: “probiotics” or “lactobacillus” or “bifidobacterium” or “bacteriotherapy” or “fermented milk” or “lactic acid bacteria” and “supplement” or “treatment” and “allergy” or “respiratory allergy” or “asthma” or “allergic rhinitis” and “children” or “pediatric” or “adults” and “clinical trial” or “randomized controlled trial.” We then combined all the searches and retrieved the relevant articles. 

### 2.3. Data Collection and Analysis

#### 2.3.1. Methodological Quality

Each included study was evaluated with the (previously validated) 5-point Jadad scale to assess quality of trials by two independent reviewers (Table 1) [22]. This scale assigns points as follows.

1. Was the study described as randomized? (0 = no; 1 = yes)
2. Was the study described as double blind? (0 = no; 1 = yes)
3. Was there a description of withdrawals and dropouts? (0 = no; 1 = yes)
4. Was the method of randomization well described and appropriate? (0 = no; 1 = yes)
5. Was the method of double blinding well described and appropriate? (0 = no; 1 = yes)
6. Deduct 1 point if methods for randomization or blinding were inappropriate.

Out of a maximum possible score of 5, studies with scores ≥ 3 are considered to be of good qualities and were included in the analysis.

#### 2.3.2. Data Collection

 Two review authors independently reviewed the results for inclusion in the analysis. Design of the trial, comparator, characteristics of study participants, number of participants, type of intervention (dose, duration), and major outcomes were evaluated. Differences about study quality were resolved through discussion. We recorded data on a prestructured data extraction form. We assessed publication bias using the Cochrane Collaboration's “risk of bias” tool [23].

#### 2.3.3. Data Synthesis

Continuous data were expressed as mean (SD), and standardized mean difference (SMD) was obtained. The data from various studies were pooled and expressed as pooled SMD with 95% confidence interval (CI). Dichotomous data were expressed as odds ratio (OR) with 95% CI. *P* value <0.05 was considered significant. A fixed effects model was initially conducted. If significant heterogeneity existed between trials, potential sources of heterogeneity were considered, and, where appropriate, a random effects model was used. Inverted funnel plot was generated for assessment of publication bias. RevMan (version 5) was used for all the analyses.

## 3. Results

**Figure 1:**
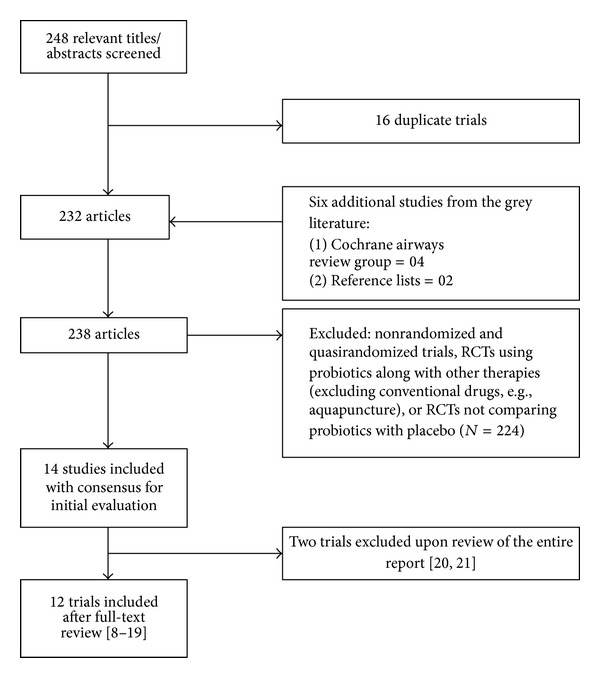
Flow diagram of search results. RCTs = Randomized controlled trials.

Ninety-two hits were obtained when the combined MeSH terms were used (Figure 1). From the initial search, 14 studies were considered as potentially eligible. These studies were further evaluated for eligibility. Twelve studies were found to be eligible for inclusion in this systematic review; two studies were excluded [8–21].** **Information on relation to methodological quality, characteristics of participants, interventions, and outcome measures of each included trial is provided in Table 1. Two studies were excluded in view of low methodological quality (Table 2). The quality of included studies was good with Jadad score ≥3. Most studies had adequate randomization and blinded intervention. Allocation concealment was unclear in all but one study [8]. Few studies measured clinically relevant outcomes separately for asthma and allergic rhinitis. Subjects with severe persistent asthma symptoms were not included, as none of the studies included participants with this symptom. For the studies in which the results were expressed as mean (95% CI) or mean ± SE, the standard deviation was derived from the available data. Twelve included studies enrolled a total of 995 participants (547 for treatment and 488 as control subjects, which totaled 682 after losses to follow-up) involving all age groups and both sexes. In 5 trials, participants were administered probiotics on/before the onset of pollen season and continued until the completion of the pollen season [9, 10, 14–16], and in other 7 trials, intervention was started at the beginning of the trial and continued for variable time periods. Three studies provided data on the assessment of quality of life and three about adverse events [11–13].

### 3.1. Primary Outcome Measure 

#### 3.1.1. Quality of Life Score at the End of Treatment in AR or Asthma

**Figure 2:**
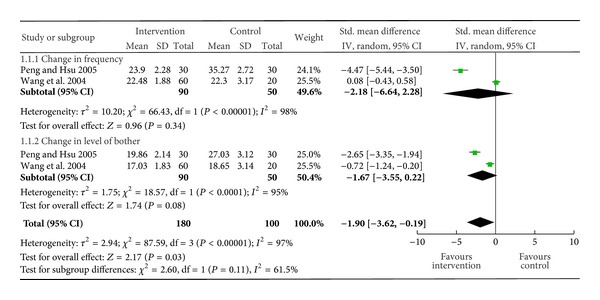
Change in quality of life score in allergic rhinitis.

Two studies evaluated the quality of life score (frequency, level of bother) in 170 patients with AR (Figure 2) [11, 12]. Compared to the placebo group, intervention group showed an improvement in the overall quality of life score (SMD −1.9 (95% CI −3.62, −0.19); *P* = 0.03), but not in the improvement of the individual (change in frequency, SMD −2.18 (95% CI −6.64, 2.28; *P* = 0.34) and change in level of bother, SMD −1.67 (95% CI −3.55, −0.22; *P* = 0.08)) score. However, as there was a high degree of heterogeneity for this outcome, the beneficial result should be interpreted with caution. Only one study reported this outcome in subjects with asthma [13]. There was no difference between the two groups when either the separate domains (activity limitations, symptoms, emotional function, exposure to environmental stimulus) or overall quality of life scores (Juniper scale) were compared.

#### 3.1.2. Secondary Outcome Measures

Due to paucity of study data, pooling could not be done for the following secondary outcomes (except for the change in blood/immunological parameters). We will discuss the result of individual study.

#### 3.1.3. Time (Months) Free from Episodes of Asthma or AR

This was measured by Giovannini et al. [8]. There was no significant difference between the two groups, with mean (95% CI) time of 6.2 (5.0 to 7.4) months in the intervention group versus 5.1 (4.0 to 6.3) months in the control group (*P* = 0.4) for asthma and mean (95% CI) time of 4.1 (3.1 to 5.0) months in the intervention group versus 3.3 (2.4 to 4.3) months in the control group (*P* = 0.9) for AR.

#### 3.1.4. Mean Duration of an Episode of Asthma

This was measured by Giovannini et al. [8]. No significant difference between intervention and control group was found, with mean difference (MD) (95% CI) −0.47 (−1.47 to 0.53) days for asthma and MD (95% CI) 1.02 (−0.27 to 2.32) days for AR. 

#### 3.1.5. Number of Episodes of Asthma or AR

This was measured by Giovannini et al. [8]. No difference between the two groups was found in case of asthma (data not given), but in case of AR, there was significant decrease in the number of episodes in intervention group, with a mean number of episodes of, respectively, 3.2 (2.4 to 4.1) versus* *4.8 (3.5 to 6.1) (*P* = 0.05), that is, an MD of 1.6 episodes/year.

#### 3.1.6. Change in Weekly Allergic Lung or Rhinitis Symptom Score

This was measured by two studies. In one study, there was no significant change between the two groups from baseline to the period after the pollen season [9]. After pollen season, the mean (95% CI) change in nasal symptom score in the intervention group was 3.7 (−0.3 to 7.7) and in the placebo group was 3.3 (−2.2 to 8.8) (*P* = 0.9). The mean (95% CI) change in allergic lung symptom score in the intervention group was 0.8 (−1.7 to 3.2) and in the placebo group was 6.8 (−0.3 to 13.9) (*P* = 0.1). In another study, probiotic treated subjects had significantly reduced eye and nose symptom scores (eye scores: 1.0 ± 0.5 versus 2.4 ± 0.9 at 8 weeks and 0.6 ± 0.3 versus 2.1 ± 0.7 at 12 weeks, *P* = 0.001 and 0.000; nasal scores: 5.1 ± 0.9 versus 6.5 ± 1.2 at 8 weeks and 3.1 ± 0.8 versus 5.1 ± 1.5 at 12 weeks, *P* = 0.001 and 0.000), but not lung symptom scores [18].

#### 3.1.7. Change in Weekly Allergy and Asthma Medication Score

This was measured by two studies. In one study, the total use of allergy and asthma medication increased more in the intervention group, but the difference did not reach statistical significance (*P* = 0.06) [9]. The mean (95% CI) increase in the intervention group was 2.7 (1.60 to 3.7) and in the placebo group was 1.2 (0.05 to 2.4). In another study, there was a statistically significant change in medication scores for rhinitis at visit 4 between the intervention and the placebo group (2.4 ± 0.9 versus 2.8 ± 1.1, *P* = 0.006) [18].

#### 3.1.8. Cumulative Number of Asthma and Rhinitis Episodes

This was measured by Giovannini et al. [8]. Though statistically not significant, the cumulative episodes were lower in the intervention than in the placebo group (median, interquartile range (IQR), 5 (2 to 9) versus 7 (4 to 11)) (*P* = 0.073).

#### 3.1.9. Time Free from Episodes of Asthma/Rhinitis

This was measured by Giovannini et al. [8]. This was significantly longer in the intervention group compared with the placebo group (mean (95% CI) 3.5 (2.7 to 4.3) versus 2.1 (1.5 to 2.7) months) (*P* = 0.027). 

#### 3.1.10. Change in the Pulmonary Function Tests

Two studies reported the result of peak expiratory flow rates (PEFR) or spirometry values. One study including the adult participants could not note any difference in mean daily peak flows or changes in spirometric values [13]. Another study including children found significant improvement in the pulmonary function tests (FEV1, FVC, FEV1/FVC(%), and MEF_25–75_) and PEFR in the intervention group [19]. 

#### 3.1.11. Changes in Blood or Immunological Parameters

**Figure 3:**
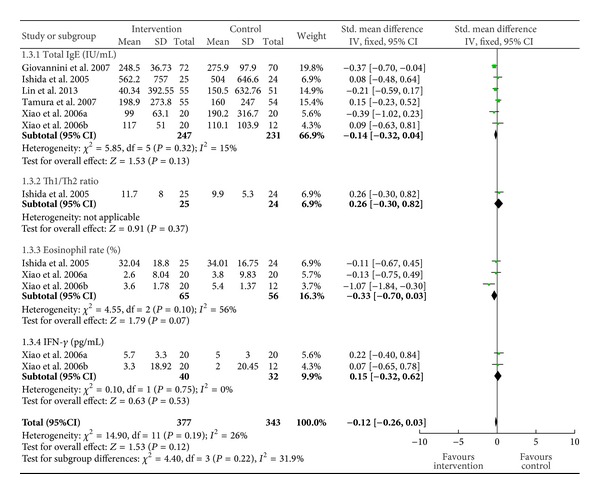
Change in the blood or immunological parameters.

Data from six studies including 720 patients were used for this analysis [8, 10, 14, 16–18]. There was no significant change in the total IgE (SMD −0.14 (95% CI −0.32), 0.04; *P* = 0.13), Th1/Th2 ration (SMD 0.26 (95% CI −0.3), 0.82; *P* = 0.37), eosinophils rate (SMD −0.33 (95% CI −0.7, 0.03); *P* = 0.07), and IFN-gamma level in the probiotic group, (SMD 0.15 (95% CI −0.32, 0.62; *P* = 0.53)) (Figure 3).

#### 3.1.12. Side Effects Noted (If Any)

None of the 12 studies reported a definition of what constituted an adverse event. Two of the 3 studies that did monitor for adverse events reported absence of adverse events [11, 12]. The third reported 14 minor adverse events (including cold, diarrhea, and vomiting) but not the group (treatment or control) in which they occurred [10].

### 3.2. Publication Bias

**Figure 4:**
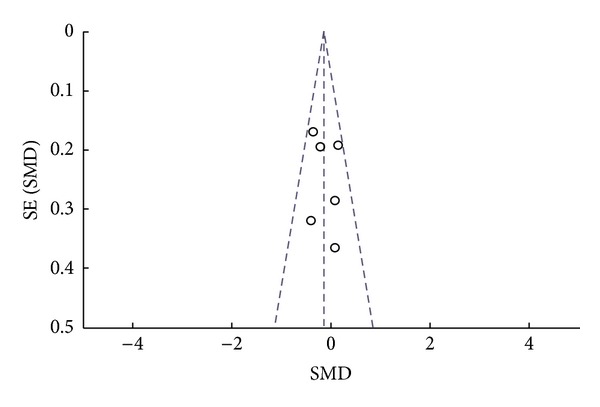
Funnel plot. Assessing publication bias using the SMD and 1/SE values from one of the tertiary outcome measures (serum total IgE level).

To assess whether there was a bias in the published literature, funnel plot was constructed using the SMD and 1/SE values obtained from studies for one of the secondary outcome measures (serum total IgE level) as there was paucity of data for primary outcome measures. In the absence of a publication bias, such a plot is expected to have a shape resembling an inverted funnel [24]. From the funnel plot generated, the possibility of publication bias in the analysis is less likely (Figure 4).

## 4. Discussion

In the present systematic review, treatment with probiotics was shown to improve the quality of life score of patients with AR (but not asthma) at the end of treatment. Other outcomes showing improvement with probiotic treatment were time in months free from episodes of asthma and of rhinitis and decrease in the number of episodes of rhinitis per year. However, the results are inconsistent (suggesting greater caution in interpretation) which we will discuss next.

Among studies included in our review, 3 studies defined primary outcome measures [8, 11, 12], 1 study defined clinically relevant outcomes (time free from episodes of asthma and/or rhinitis and the cumulative number and duration of episodes) [8], and the other 2 were based on quality of life in subjects with AR [11, 12]. Study done by Giovannini et al. was the largest one with highest quality [8]. But in this study placebo used was nonfermented milk and was poorly defined as in some of the other studies discussed later. Compliance in treatment group was good, however, based on nonconsumed pots, it was approximately 14% reduced during the second semester of intervention as compared with the first semester. This may be a limitation of the study that could have prevented finding significant differences between groups. Five studies reported effects of probiotics on allergic symptoms induced during pollen season of Japanese cedar pollen (JCP) or birch pollen in patients with history of such allergy (confirmed by symptoms as well as laboratory tests) [9, 10, 14–16]. In these trials, participants were administered probiotics on/before the onset of pollen season and continued until the completion of the pollen season. BB536-supplemented yogurt has been demonstrated to have a pronounced promoting effect on intestinal environments after 2 weeks of intake at a dose of 100 g per day [25]. For this reason, in these studies, probiotics were administrated before pollen exposure. However, in one trial, no beneficial effect in the probiotic group was demonstrated instead of starting the treatment 2.5 months before the birch-pollen season [9]. In this trial, the period was certainly long enough to have an effect on the microbial flora, but beneficial changes in immune responses may take longer as the probiotic strain was different. Another important reason is the difference regarding the validity of the clinical effects of lactic acid bacteria among species and strains. In fact, in vitro studies using human mononuclear cells have indicated that there are strain-dependent differences in the ability of lactic acid bacteria to induce immunoregulatory monokines such as interleukin-12 [26]. Contribution of the species- and strain-specific nature of lactic acid bacteria on the efficacy of improving allergic symptoms should be considered. 

The sample size or the number of subjects enrolled varied markedly between the studies (with most studies having small sample size) and may have increased the chance of type II error. In one study, the increase in total symptom score from baseline to pollen season was +25.9 in the intervention and +28.0 in the placebo group [9]. The sample size in this study should have been 1500 subjects per group to show the achieved treatment difference of −2.0 in total symptom score to be statistically different at the level of 5% and power of 80%. Furthermore, in the same study, there was a greater increase in the use of allergy and asthma medication from the baseline to the pollen season in the intervention group compared with the placebo group. This difference almost reached significance (*P* = 0.06) in spite of the small numbers.

There were 2 crossover studies included in the current review. First one by Xiao et al. found that BB536 is effective in relieving symptoms of JCP allergy, and scores for disruption of normal activities were significantly lower in the BB536 group compared with the placebo [16], whereas those done by Wheeler et al. found no differences in mean daily peak flows or changes in spirometric values and quality of life scores [13]. Though crossover studies allow maximal opportunity for the revelation of an enhancing effect of probiotics, they have their inherent problems, and caution should be exercised in interpretation of their results. The major drawback in the later study was very small sample size, which could have masked the beneficial effect of probiotic bacteria.

Placebo was poorly defined in most of the studies. Many studies used nonfermented milk or plain yogurt as placebo. A better control would have been fermented milk without the addition of the probiotic bacteria or sterilized fermented milk [27]. The studies demonstrated the effect of fermented milk containing a specific probiotic strain, but it is not possible to conclude the effect of probiotic bacteria per se. Indeed, studies state that plain yoghurt has some antiallergic effect and may have an impact on rhinitis and asthma [28, 29]. 

All these trials have used different doses and durations as well as different strains of probiotics. The studies on asthma have used only *Lactobacillus* (sp: *acidophilus*, *rhamnosus*, *casei*) as the probiotic strain, whereas studies on allergic rhinitis have used *Bifidobacterium* (sp: *longum*) as well as *Lactobacillus* strains. In all the trials, the minimum dose of probiotics administered was >5 billion colony forming unit (CFU), and minimum duration of administration was 1 month. It has been hypothesized that some probiotic strains and/or their fermentation products are responsible for improvement of allergic rhinitis and that the immunostimulatory effect of *Lactobacillus* may be dose dependant [9, 16, 20, 25]. 

The effects of probiotics to modulate blood/immunological parameters associated with allergic symptoms should be elucidated as some studies found beneficial effect on clinical parameters without significant change in the immunological parameters. In this meta-analysis, we found no significant overall change in immunological parameters in the probiotics group. Probiotics may possibly improve subjective symptoms even if immunological parameters such as the allergen-specific IgE level or Th1/Th2 imbalance are not normalized. Involvement of natural killer T-cells and regulatory T-cells in the induction and control of allergic responses has been proposed [30, 31]. Therefore, other mechanisms beside suppression of IgE production or normalization of Th1/Th2 imbalance could be involved in the antiallergic activity exerted by probiotics in humans.

It is well known that systematic reviews are associated with limitations, and the results obtained with these methods should be analyzed accordingly. The numbers of patients analyzed were small to reflect the data on the whole population. Twelve controlled trials included a total of 995 subjects of both age and sex with a paucity of clinically relevant outcome measures. Only one trial was of good quality, and the other nine trials were more or less well designed. There was no uniformity in definition of respiratory allergic asthma and rhinitis symptoms for selection of subjects for probiotics in these trials. Even though we tried to include only those subjects with pure respiratory allergy at the time of study enrolment, we cannot be absolutely sure about the contamination of groups. Indeed, this meta-analysis highlights the paucity of good quality clinical trials evaluating the role of probiotics when used as treatment, either alone or in combination with medications used to control symptoms of respiratory allergies. 

## 5. Conclusion 

As the current evidence was generated from few trials with high degree of heterogeneity, routine use of probiotics as an additive on therapy in subjects with allergic airway diseases cannot be recommended.

## Figures and Tables

**Table 1:** Characteristics of included studies in the review.

Study (year)	Methods	Target population	Intervention (probiotic strain)	Outcomes	Notes
Giovannini et al. (2007) [8]	Randomized placebo-controlled, double-blind trial	187 children aged 2–5 yrs	*Lactobacillus bulgaricus, Streptococcus thermophilus*, and *Lactobacillus casei*. 100 mL/day for 12 months	Number of days free from and cumulative number and duration of episodes of asthma and/or rhinitis	Exclusion criteria—cow's milk or food allergy, lactose intolerance, chronic disease, perinatal respiratory problems, antibiotic use in the preceding 4 wk before starting intervention

Helin et al. (2002) [9]	Randomized placebo controlled double blind trial	36 subjects aged 14–36 yrs	*Lactobacillus rhamnosus*. 4 capsules/day for 5.5 months	Changes in allergic nose, eye, lung, and total symptom scores	Exclusion criteria—other pollen allergies, smoking, pregnancy and lactation, use of immunotherapy or long-term medication or antibiotics and probiotic products

Tamura et al. (2007) [10]	Randomized placebo controlled, double blind trial	120 subjects aged 39.3 ± 8.0 years (probiotic) and 39.5 ± 10 (placebo)	*Lactobacilluscasei shirota*. 80 mL/day for 8 weeks	Change in symptom medication score (SMS)	Exclusion criteria—use of antihistamines or antiallergic medication, upper respiratory tract infection or polyp, asthma, severe chronic systemic disorder, hyposensitization therapy, cow's milk allergy, drinking dairy products containing lactic acid bacteria

Peng and Hsu (2005) [11]	Randomized placebo controlled, double blind trial	90 subjects aged 16.07 ± 2.11 yrs (live probiotic), 14.50 ± 1.78 (heat-killed probiotic), 16.60 ± 2.02 (placebo)	*Lactobacillus paracasei*. 2 capsules/day for 30 days	Change in modified pediatric rhinoconjunctivitis quality of life score	Exclusion criteria—steroid treatment, neuropsychiatric disease or congenital immunodeficiency, probiotic allergy

Wang et al. (2004) [12]	Randomized placebo controlled double blind trial	80 children aged 15.87 ± 1.53 yrs (probiotic) and 14.00 ± 1.90 (placebo)	*Lactobacillus* * paracasei*-33. 200–400 mL/day for 30 days	Change in modified pediatric rhinoconjunctivitis quality of life score	Exclusion criteria—pregnancy, steroid treatment, smoking, neuropsychiatric disease or congenital immunodeficiency or cow's milk allergy

Wheeler et al. (1997) [13]	Randomized crossover design	15 adult patients aged 13 to 45 years	*Lactobacillus* *acidophilus*, *Lactobacillus Bulgaricus*, and *Streptococcus thermophilus*. 450 g/day for 2 months	Immune and clinical parameters including pulmonary function tests and quality of life assessments	Exclusion criteria—smoking history, on antibiotics, receiving immunotherapy

Xiao et al. (2006a) [14]	Randomized placebo controlled, double blind trial	40 adult subjects aged 23–61 yrs (probiotic) and 24–55 yrs (placebo)	*Bifidobacterium longum*536 (BB536). 200 g/day for 14 weeks	Effect on subjective symptoms score	Exclusion criteria—subjects with extreme severe symptom of JCPsis

Xiao et al. (2006b) [15]	Randomized placebo controlled, double blind trial	44 adult subjects aged 22 to 48 yrs (placebo) and 26 to 57 yrs (probiotic)	BB536 powder twice daily for 13 weeks	Effect on subjective symptom scores	Exclusion criteria—subjects with extreme severe symptom of JCPsis

Xiao et al. (2007) [16]	Randomized crossover design	24 adult subjects aged 41.0 ± 8 yrs (group-A) and 37.6 ± 7 yrs (group-B)	BB536 powder twice a day for 8 weeks	Effect on symptom and medication score	No exclusion criteria stated

Ishida et al. (2005) [17]	Randomized placebo controlled, double blind trial	49 adult subjects aged 34.0 ± 3.4 yrs (intervention) and 36.9 ± 3.0 yrs (placebo)	*Lactobacillus acidophilus* L-92. 100 mL/day for 8 weeks	Change in SMS (both nasal and ocular) values	No exclusion criteria stated

Chen et al. (2010) [18]	Randomized placebo controlled, double blind trial	109 children aged 6–12 years	*Lactobacillus salivarius* 4 × 10^9^ colony forming units/g/day as a powder mixed with food or water for 12 weeks	The scoring allergic rhinitis index (specific symptoms scores and SMS and blood parameters	Those treated with immunotherapy and those with recurrent respiratory tract and infectious diseases were excluded

Lin et al. (2013) [19]	Randomized placebo controlled, double blind trial	105 children aged 6–12 years	*Lactobacillus gasseri* A5 capsule (2 × 10^9^ cells/capsule) twice a day for 2 months	Peak expiratory flow rates, symptoms of asthma, and AR scores of the patients, immunological parameters	Those treated with immunotherapy, corticosteroids, and inhaled b2-agonists, anatomical abnormality of the upper respiratory tract and congenital cardiovascular diseases were excluded

**Table 2:** Characteristics of studies excluded from the review.

Study	Reason for exclusion
Trapp et al. 1993 [20]	Groups 1 and 2 randomized and double blinded, but group 3 were those who did not want to eat yogurt.

Ishida et al. 2005 [21]	Randomized single-blind study with quality score = 0.
